# Using lymph node swelling as a potential biomarker for successful vaccination

**DOI:** 10.18632/oncotarget.9580

**Published:** 2016-05-24

**Authors:** Kimberly D. Brewer, Drew R. DeBay, Iulia Dude, Christa Davis, Kerry Lake, Cathryn Parsons, Rajkannan Rajagopalan, Genevieve Weir, Marianne M. Stanford, Marc Mansour, Chris V. Bowen

**Affiliations:** ^1^ Biomedical Translational Imaging Centre (BIOTIC), Halifax, NS, Canada; ^2^ Department of Radiology, Dalhousie University, Halifax, NS, Canada; ^3^ Department of Physics, Dalhousie University, Halifax, NS, Canada; ^4^ Immunovaccine Inc., Halifax, NS, Canada; ^5^ Department of Microbiology and Immunology, Dalhousie University, Halifax, NS, Canada; ^6^ School of Biomedical Engineering, Dalhousie University, Halifax, NS, Canada

**Keywords:** magnetic resonance imaging, cancer, vaccines, biomarker, emulsion, Immunology and Microbiology Section, Immune response, Immunity

## Abstract

There is currently a lack of biomarkers to help properly assess novel immunotherapies at both the preclinical and clinical stages of development. Recent work done by our group indicated significant volume changes in the vaccine draining right lymph node (RLN) volumes of mice that had been vaccinated with DepoVax^TM^, a lipid-based vaccine platform that was developed to enhance the potency of peptide-based vaccines. These changes in lymph node (LN) volume were unique to vaccinated mice.

To better assess the potential of volumetric LN markers for multiple vaccination platforms, we evaluated 100 tumor bearing mice and assessed their response to vaccination with either a DepoVax based vaccine (DPX) or a water–in-oil emulsion (w/o), and compared them to untreated controls. MRI was used to longitudinally monitor LN and tumor volumes weekly over 4 weeks. We then evaluated changes in LN volumes occurring in response to therapy as a potential predictive biomarker for treatment success.

We found that for both vaccine types, DPX and w/o, the %RLN volumetric increase over baseline and the ratio of RLN/LLN were strong predictors of successful tumor suppression (LLN is left inguinal LN). The area under the curve (AUC) was greatest, between 0.75-0.85, two (%RLN) or three (RLN/LLN) weeks post-vaccination. For optimized critical thresholds we found these biomarkers consistently had sensitivity >90% and specificity >70% indicating strong prognostic potential. Vaccination with DepoVax had a more pronounced effect on draining lymph nodes than w/o emulsion vaccines, which correlated with a higher anti-tumor activity in DPX-treated mice.

## INTRODUCTION

Immunotherapies comprise one of the most important and fastest growing classes of cancer therapies, with *Science* magazine naming cancer immunotherapy “the breakthrough of the year” in 2013 [1]. There have been several clinical successes over the last few years, with several monoclonal antibodies, cytokines, three checkpoint inhibitors and a therapeutic vaccine being approved for clinical use [2–6]. The recent success of checkpoint inhibitors in particular has reinvigorated the field, resulting in fresh enthusiasm for a variety of new immunotherapies, and especially in combining therapies to maximize clinical response. However, there remains a lack of validated biomarkers to help properly assess new therapies at both the preclinical and clinical stages of development.

The need for more accurate biomarkers to assist with therapeutic development and clinical translation is so strong that, in a report issued by the FDA in 2006, they stressed the need for not only the development of more safety biomarkers (used to evaluate toxicity and biocompatibility), but biomarkers that could be used to advance personalized medicine and qualify surrogate endpoints of treatment success or failure [7]. Qualifying new biomarkers was identified in the report as one of the main components that were necessary for more successful clinical translation of therapies. The development of biomarkers for cancer therapies has progressed but continues to be an important area of research [8, 9].

There are a number of histopathological biomarkers currently under study in a number of preclinical and clinical trials. Recently, there has also been a significant amount of research conducted evaluating and quantifying the amount and type of immune cells infiltrating certain cancer types, and using that as a predictor of host response to cancer [10–13]. This biomarker is known as the “immunoscore” and is a direct measure of pre-existing immunological activity at the tumor site. Yet it requires use of biopsy tissue, which is an invasive technique and is not available for all tumors.

In addition to immune infiltrates, the expressions of specific immune system markers have also been explored as biomarkers. One example of this is quantitation of the expression of PD-L1 within tumors [14] as a prediction of checkpoint therapy success. There have been several trials showing a positive correlation between PD-L1 expression and response to anti-PD-1 therapy (see Patel et al. [14] for a review of several studies). However, its use as a predictive biomarker is confounded by several issues including tissue preparation [14], primary *versus* metastatic biopsies [15] and intratumoral heterogeneity [16].

Other potential biomarkers include the use of EGFR mutations [17], SUMO pathway components [18], and genomic characteristics of tumors [19, 20], to name but a few. However, all of the above-named biomarkers require biopsy tissue for either histological or genetic analysis, and many require primary tumor samples for analysis. There remain significant questions about their feasibility in larger populations. Unfortunately, there is not always a primary tumor site available for resection and analysis, and many of these techniques do not necessarily predict the effects on metastases, or how tumors change and respond to primary treatments (including chemotherapies), nor do they allow for longitudinal assessment of treatment success. While some blood-based biomarkers have been proposed, particularly the use of circulating tumor cells (CTCs) and microRNA [21, 22], these biomarkers are often limited by the amount needed for successful diagnosis. Importantly, there is need for a test that can be used to indicate early response to treatment.

Imaging-based biomarkers are more desirable for a number of reasons. Imaging is commonly done as standard patient care and as part of clinical trials. It is also increasingly done in preclinical studies, allowing for more efficient translation of therapies from the bench to the clinic. Additionally, imaging often allows for repeated longitudinal evaluation and follow-up, which enables clinicians to potentially adjust and personalize each patient's standard of care based on their responses.

The Response Evaluation Criteria in Solid Tumors, or RECIST 1.1 [23, 24] is currently the standard of care for evaluating treatment success. While RECIST has proven to be an excellent indicator of chemotherapeutic success, it is a poor biomarker for evaluating the new class of targeted therapies, including biologics, immunotherapies, and other combined therapies [25]. Due to the increased prevalence and demonstrated potential of immunotherapeutic drugs, there has been a push by clinicians and pharmaceutical companies to adjust the RECIST criteria to improve evaluation of these therapies.

A novel set of criteria, called immune-related response criteria (irRC), evaluating the total tumor burden, has been proposed [26, 27]. These criteria have been tested in a Phase 2 clinical trial for a recently approved monoclonal antibody checkpoint inhibitor, ipilimumab (Yervoy) in melanoma [28], and are being increasing studied by a number of sites. However, even the irRC criteria rely on monitoring volumetric changes at the tumor itself, and so are decidedly indirect with respect to changes in tumor immune responses, and are not adequate for measuring improved overall survival that may be facilitated by immunotherapies irrespective of their effects on tumor volumes.

There has also been a gradual shift to biomarkers using metabolically-based positron emission tomography (PET), replacing those of anatomically-based computed tomography (CT) for evaluating novel cancer therapy responses. PET-based criteria similar to RECIST called PERCIST have been proposed [29], which could be more useful for evaluating immunotherapeutics. The proposed PERCIST criterion measures the 2-deoxy-2-(^18^F) fluoro-D-glucose (18FDG) response at the tumor site to evaluate metabolic changes, and then combines these with tumor volumetric changes to determine response to therapy. Other PET tracers are also being developed for evaluating immunotherapies, including [^18^F]-2-fluoro-D (arabinofuranosyl)cytosine (18FAC) and 3′-deoxy-3′[18F]fluorothymidine (18FLT) [30, 31]. While more direct, these potential biomarkers have not yet been extensively tested or evaluated, and face several barriers to implementation, particularly the use of clinical PET software to automatically calculate standardized uptake values (SUV) in the liver, and obtain glycolysis measures [29].

In recent work by our group [32–34] we observed significant changes in the vaccine draining lymph node volumes (RLN) of mice that had been vaccinated with DepoVax^TM^ (DPX), a lipid-based vaccine platform that was developed to enhance the potency of peptide-based vaccines. DPX-based cancer immunotherapeutics are currently in Phase 2 clinical development [35, 36]. The changes in lymph node volume we observed were unique to vaccinated mice, and likely indicative of clonal expansion of effector T-cells, previously demonstrated with DPX vaccines [37]. In a study with a smaller group of mice (*n* = 21), an early increase (12-19 days post-treatment) in the vaccine-draining right inguinal lymph node (RLN) correlated with overall therapy success (tumor suppression 6 weeks post-therapy). This indicated the potential of RLN for use as a predictive biomarker early in therapy. Additionally, increases in lymph node volume are a common side effect of many clinical vaccines [38, 39], indicating a high possibility of clinical translatability.

In this work we evaluated over 100 tumor bearing mice and assessed their response to vaccination with either a DPX based vaccine or a water-in-oil emulsion, and compared them to untreated controls. The water-in-oil emulsion was used as most other cancer vaccines in testing that use oil do so in an emulsion format, making it consistent with other vaccine adjuvants in development, such as MF59, ASO3 and AFO3 [40]. Magnetic resonance imaging (MRI) was used to longitudinally monitor lymph node and tumor volumes weekly over 4 weeks. We then evaluated changes in lymph node volumes occurring in response to therapy as a potential predictive biomarker for treatment success.

## RESULTS

**Figure 1:**
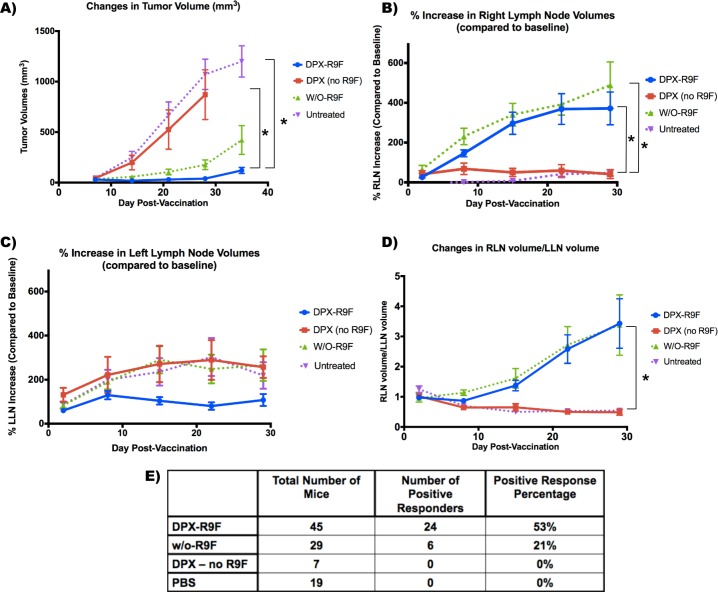
Graphs demonstrating volumetric changes in inguinal lymph nodes and tumors over the course of the study for all mice (*N* = 100) **A.** Tumor volume timecourse (mm^3^). **B.** % Right lymph node (RLN) volume increase over time (draining vaccine site). **C.** % left lymph node (LLN) volume increase over time (draining tumor site). **D.** Ratio of RLN volume over LLN volume. **E.** Total number of each mice per group, and the number of mice that had successful tumor suppression (positive responders). Success was defined as tumors being less than 50 mm^3^ by the end of the study. * indicates that groups were significantly different (*p* < 0.05) at the end of the study. Data on graphs is mean ± SE.

These results represent the accumulation of tumor and lymph node volumetric data from 100 mice in six different studies. As described in the methods, all 100 mice underwent C3 tumor cell implantation with 5×10^5^ cells implanted subcutaneously (s.c.) into the left flank. Five days post-implantation, mice received either i) DPX-R9F, ii) DPX (no R9F), iii) water/oil (w/o-R9F), or iv) PBS control injection (see methods for more details). A subset of this data with 21 mice that were untreated, treated with DPX (DPX-R9F) or treated with a vehicle control injection (DPX-no R9F) was previously published [34]. Figure 1 demonstrates the extent of change in volume in both the left and right lymph nodes (LLN and RLN, respectively) in response to vaccination for both vaccine types and the control groups, as well as the changes in tumor volume over the length of the tumor challenge.

Tumor volumes are displayed in Figure 1a with both vaccine formulations causing substantial tumor suppression compared to the control groups. A successful, or “positive” response was registered if the tumor volume was determined (using MRI) to be less than 50mm^3^ at the end of the study. This metric was chosen based upon when these particular tumors became measurable using traditional caliper techniques. Tumors < 50mm^3^ could generally be felt only *via* palpation (or potentially viewed with MRI), but could not necessarily be measured. Therefore, if a tumor became “measurable” *via* caliper, it was considered a failure, or “negative” response. Given this metric, 53% of DPX-R9F mice had positive responses, *versus* only 21% of w/o-R9F mice and neither the PBS nor the DPX no R9F groups had any positive responders (see Figure 1e).

A mixed ANOVA indicated that there were significant main effects (F(1.639,139.349) = 110.217, *p* < 0.001) and that there was also a significant interaction between group and study day (F(4.9,139.349) = 34.589, *p* < 0.001). We therefore did a separate repeated-measures ANOVA for each group to assess within-subject differences and a separate univariate ANOVA for days 8, 15, 22 and 29. From day 15 onward, both w/o-R9F and DPX-R9F tumor volumes were significantly different from both control groups. The control (PBS) group was the only group to have statistically significant changes in tumor volume at each time point (*p* < 0.05), although the w/o-R9F group tumor volumes were significantly higher at day 29 compared to day 8. The repeated measures ANOVA for the DPX (no R9F) group exhibited significant effect (*p* < 0.01), however no pairs were found to be significant after multiple comparison corrections.

As previously seen [34], both the DPX-R9F and w/o-R9F groups exhibit extremely large volume increases in the RLN (i.e. vaccine-draining) by two weeks post-vaccination (see Figure 1b). In contrast, both the PBS control group and the DPX-no R9F groups exhibit smaller changes in volume, with the PBS group in particular exhibiting no significant increases until between 21 and 28 days post-vaccination (i.e. 26 and 33 days post-implantation). A mixed ANOVA analysis found statistically significant effects (F(1.368,64.296) = 5.827, *p* < 0.01), with no interaction effects between group and study day (F(4.104, 64.296) = 1.949, *p* > 0.05). Post-hoc tests revealed that both DPX-R9F and w/o-R9F were significantly different than PBS (*p* < 0.05), and that day 2 was significantly different than days 8, 15 and 22 (*p* < 0.05).

For the LLN in Figure 1c (i.e. tumor-draining), DPX-R9F mice have smaller volume increases than all of the other groups, likely due to the improved overall tumor suppression, as seen in Figure 1a. A mixed ANOVA did indicate significant main effects over time (F(1.908,89.662) = 4.461, *p* < 0.05) and no interaction effects (F(5.723,89.662) = 1.872, *p* > 0.05). There were no significant differences between groups, overall, day 29 was significantly different from day 2 and day 22 (*p* < 0.05).

The RLN/LLN volume ratio (Figure 1d) increases with time for both w/o-R9F and DPX-R9F groups, with the largest differences between vaccinated and control mice occurring between 28 to 35 days post-vaccination. However, although the differences between the vaccinated and control groups appear large, a mixed ANOVA found that there were no significant main effects over time (F(1.444,66.445) = 3.102, *p* > 0.05) or interaction effects (F(4.333,66.445) = 1.417, *p* > 0.05), however there were significant effects between groups, with post-hoc testing showing both untreated, i.e. the PBS and the DPX-no R9F groups, being significantly different from the “treated groups”, i.e. the DPX-R9F and w/o-R9F groups (*p* < 0.05).

**Figure 2:**
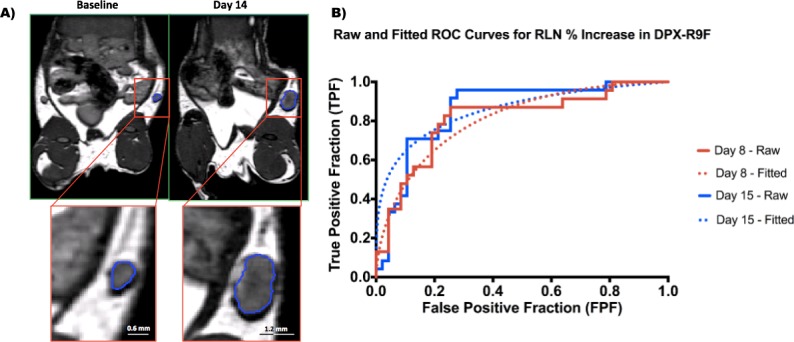
Changes in right lymph node volumes **A.** BSSFP MRI images (150um)^3^ isotropic voxels of a representative mouse from the DPX group. The segmented right lymph node (RLN) can be seen in close up in the lower left panel. This particular lymph node swelled from 1.41 mm^3^ at baseline to 15.91 mm^3^ 14 days post-injection. **B.** ROC curves generated for the % RLN increase at day 8 and day 15 (DPX subset). The solid lines indicate the ROC curves generated from the raw data, and the dotted lines indicate the fitted model generated by ROCkit.

Representative MR images demonstrating these large RLN volumetric changes are seen in Figure 2a. Given these significant changes in the RLN (*p* < 0.05), we therefore concentrated on testing potential biomarkers using the RLN. Figure 2b demonstrates the ROC curves generated using the raw data and the fitted curves generated using ROCkit. As expected, the fitted curves in Figure 2b extrapolate a larger number of data points and are therefore much smoother, however both accurately represent the ROC behaviour.

**Figure 3:**
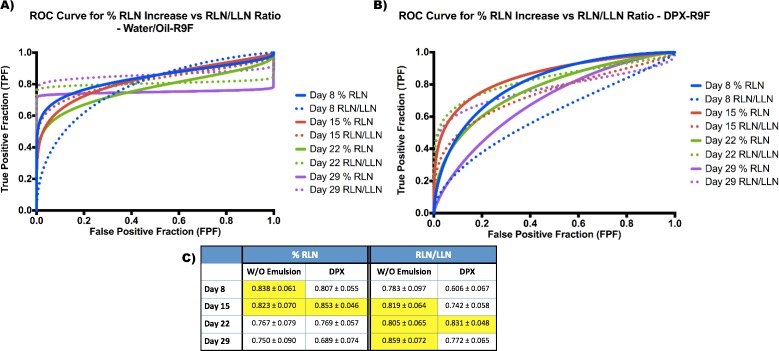
ROC Curves for Biomarkers in both DPX and water/oil emulsion groups **A.** Fitted ROC curves for each time point comparing both biomarkers in the water/oil emulsion group. **B.** Fitted ROC curves for each time point comparing both biomarkers in the DPX group. **C.** Area under the curve (AUC) values for each biomarker in each group at each time point. Optimal values are highlighted in yellow.

**Figure 4:**
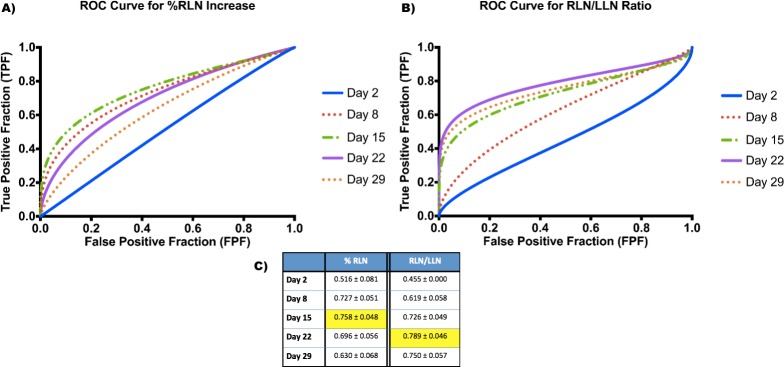
ROC Curves for Biomarkers generated for all mice **A.** Fitted ROC curves for each time point for % RLN increase biomarker. **B.** Fitted ROC curves for each time point for RLN/LLN ratio biomarker. **C.** Area under the curve (AUC) values for each biomarker at each time point. Optimal values are highlighted in the table.

Separate ROC curves were generated using both % increase in RLN and the volumetric ratio of RLN/LLN (Figure 3 and Figure 4). As described in the methods, the criteria for “positive suppression” was a final tumor volume < 50 mm^3^. The area under the curve (AUC) was generated for each time point and prospective biomarker as a measure of potential success.

The ROC curves for both biomarkers for the DPX subset (including DPX-R9F, DPX no R9F, PBS) are shown in Supplementary Figure 1. When using % RLN increase as a biomarker, day 15 had the highest AUC (0.853, Supplementary Figure 1c) and was the most sensitive and specific. Day 8 also had an AUC > 0.8, indicating a strong potential biomarker. However, the ratio of RLN/LLN volumes had higher AUC values (Supplementary Figure 1c) at later time points, with day 22 having the highest AUC (0.831), followed closely by day 29 and day 15.

We then compared the biomarkers for both DPX and the w/o emulsion. Generally, the ROC curves in the w/o emulsion subset (w/o-R9F and PBS) had stronger sensitivity values at higher specificities (Figure 3a), although the AUC values (Figure 3c) were similar for both the w/o emulsion and DPX subgroups (Figure 3a, 3b). Generally, for the % RLN increase, the highest AUC values were always earlier, around day 8 and day 15 post-vaccination (AUC > 0.8 at both time points for both w/o emulsion and DPX). However, AUC of the RLN/LLN ROC curves general peaked later, usually around day 22 or day 29 post-vaccination, although the day 15 AUC value was sometimes not much lower (see RLN/LLN day 15 for w/o emulsion).

We then combined data from all mice and generated ROC curves for each time point for both % RLN increase and the RLN/LLN ratio (Figure 4a, 4b). Although the overall AUC values were lower (Figure 4c), they were still in the 0.7-0.8 range for day 8 and day 15 for % RLN and days 15, 22 and 29 for RLN/LLN. As for the subsets of mice, the optimal ROC curves for all mice appeared to be day 15 for % RLN (0.758) and day 22 for RLN/LLN (0.789), indicating that these time points appear to be optimal for use as a predictive biomarker.

Using these optimal time points, we then returned to the ROC curves to choose the optimal critical threshold, from Youden's J statistic, for each biomarker (Table 1). The critical thresholds were generally similar between the DPX and w/o emulsion subsets, however the w/o emulsion critical thresholds were consistently lower, likely due to there being a much lower number of positive responders and smaller n (see Figure 1e). For all mice, using the % RLN increase, the critical threshold was a 102% increase at day 8, and a 74% increase at day 15. Using the RLN/LLN ratio, the critical threshold was 0.65 at day 15, 0.98 at day 22, and 1.33 at day 29. The corresponding sensitivity and specificity for each critical threshold can also be found in Table 1. For the two most optimal time points, day 15 for % RLN and day 22 for RLN/LLN, the critical thresholds corresponded to 97% sensitivity/55% specificity and 93% sensitivity/71% specificity. While the use of both predictive biomarkers generated excellent sensitivity, the specificity was higher for RLN/LLN.

**Table 1:** Cut-off Thresholds for Biomarkers

	% Increase in RLN *(Sensitivity, Specificity)*		RLN/LLN Change *(Sensitivity, Specificity)*		
	Day 8	Day 15	Day 15	Day 22	Day 29
DPX-R9F	76% *(87%, 74%)*	74% *(96%, 72%)*	0.93 *(75%, 69%)*	0.98 *(92%, 77%)*	1.33 *(81%, 77%)*
Water/Oil-R9F	113% *(100%, 71%)*	90% *(100%, 57%)*	0.78 *(100%, 64%)*	1.51 *(100, 77%)*	2.71 *(100%, 81%)*
All	102% *(80%, 67%)*	74% *(97%, 55%)*	0.65 *(90%, 55%)*	0.98 *(93%, 71%)*	1.33 *(84%, 75%)*

The optimal cut-off threshold for each group (for optimal time points) was calculated by maximizing Youden's index. For all mice, using the % RLN increase, the best sensitivity and specificity are achieved by using a 74% cutoff at day 15. For the RLN/LLN ratio, the best sensitivity and specificity are obtained at day 22 using a 0.98 cutoff.

**Figure 5:**
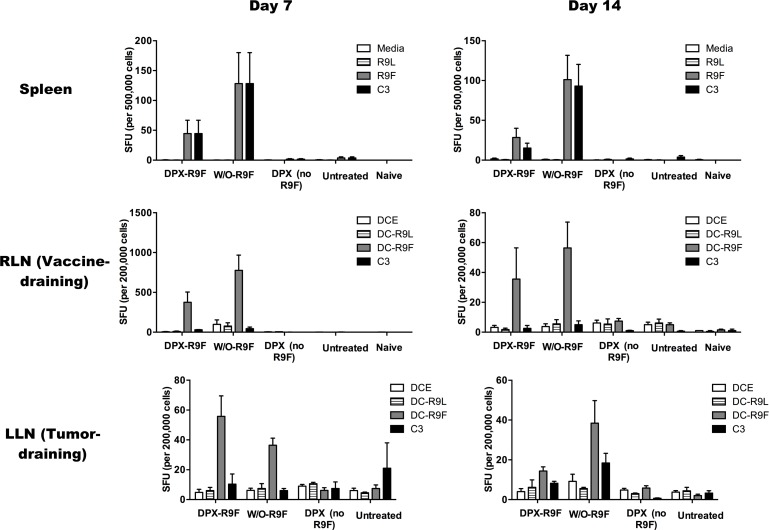
IFN-γ ELISPOT results Mice were implanted with C3 tumors and vaccinated 5 days later. Half of the mice in each group were terminated 7 days after vaccination, and the remaining at 14 days post vaccination. An IFN-γ ELISPOT was performed at the day of termination. Results are for 40 mice (*n* = 5/group/timepoint). Spleen cells were stimulated with either pure media, irrelevant peptide (R9L), relevant peptide (R9F) or C3 cells). Lymph node cells were stimulated with either DCs that were empty (DCE) or primed with irrelevant (DC-R9L) or relevant (DC-R9F) peptides or with C3 cells. All vaccinated mice had strong responses to relevant peptides at both day 7 and day 14.

**Figure 6:**
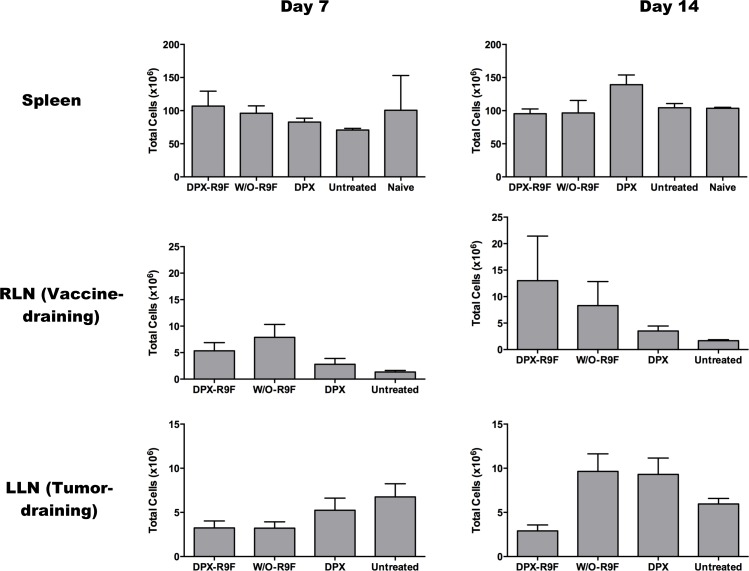
Absolute Cell Counts Absolute cell counts obtained from IFN-γ ELISPOT experiment at days 7 and 14. Results are for 40 mice (*n* = 5/group/timepoint).

Since significant swelling of the w/o emulsion group occurred at 2 weeks post vaccination and immune responses to vaccination typically peak by one week post vaccination, we evaluated immune responses in the spleen, RLN and LLN in mice at these two time points by IFN-γ ELISPOT (Figure 5). In the spleen, vaccination with either DPX-R9F or w/o-R9F elicited a strong antigen-specific immune response, untreated and vehicle control mice generated no significant responses over background. There were no significant changes in immune responses in the spleen between day 7 and day 14. In the RLN, both DPX-R9F and w/o-R9F elicited strong responses, although the response was reduced at day 14. Again, both DPX-R9F and w/o-R9F emulsion elicited significant immune responses in the LLN at day 7, while the responses generated at day 14 were detectable, but not much higher than controls. Additionally, the immune responses generated in the RLN by both DPX-R9F and w/o-R9F at day 7 were significantly larger than those generated in the LLN. The immune cell counts (Figure 6) from the ELISPOT experiment also demonstrated higher cell counts for both DPX-R9F and w/o-R9F than both control groups. The increases in LLN volumes also correlate with the total immune cell counts obtained in the ELISPOT experiment (Figure 6), with w/o-R9F having increased cell counts at day 14 compared to day 7.

## DISCUSSION

### Lymph node swelling - developing a prospective biomarker

The extensive change in volume that was observed in the inguinal LN that drains the vaccine site (RLN) has incredible promise for use as a personalized biomarker for the immunogenic effect and potentially the therapeutic success of cancer vaccines. The swelling in the RLN was significantly larger in treated mice *versus* controls and over time. However, LN volume changes in response to large tumors are commonly observed in human cancers and may be the result of increased antigenicity of large tumors stimulating the immune system (trends observed in Fig 1c in untreated groups for example). As this swelling is present even as tumors continue to grow, this response is clearly ineffective at controlling tumor growth [38]. Since advanced tumors can produce several mediators of inflammation to induce immune suppression, which can result in regional LN swelling [39], we wanted to ensure that LN swelling due to therapy could be used as a specific biomarker.

We therefore propose two potential biomarkers: one using only the localized response to vaccination (% RLN increase), whereas the other normalizing the presumably therapy-driven RLN response by any systemic tumor-driven swelling (RLN/LLN ratio). The latter ratio was proposed previously by our group in a smaller study [34]. Given that these biomarkers have the potential for clinical translation, we evaluated them using ROC curves, with the AUC value serving as a metric for overall biomarker success. These ROC curves are often used for evaluating diagnostic tests [41], particularly the AUC values, although there is not necessarily a consensus on what is considered a clinically relevant AUC. For example, a large scale study of computer-aided diagnostic tools used with digital mammography yielded an AUC of 0.83, which was considered acceptable [42].

While both potential biomarkers yielded ROC curves and AUC values indicating excellent prognostic potential, they each had very different temporal dynamics. The % RLN increase was most accurate earlier in the study, either 8 or 15 days post-vaccination. The opposite effect was seen for the RLN/LLN ratio, where this biomarker had highest AUC values, and therefore better potential for prognosis, at later time points in this study, between 15-29 days post-vaccination, often peaking at day 22. The delay in the RLN/LLN ratio response is likely due to the late time point increases in the LLN volume, likely linked to antigenicity from insufficient tumor suppression. This is supported by results seen in the DPX subgroup, which had the best tumor suppression response rate, and also the smallest increases in LLN volumes at later time points.

In order to better understand the changes in lymph node swelling and their context within the broader immune response, we did an additional ELISPOT experiment to evaluate the IFN-γ responses in the spleen and both LNs at both day 7 and day 14 post-vaccination. As expected, we found that there were strong antigen-specific immune cell responses in both the spleen and RLN at both time points for the DPX-R9F and w/o-R9F treated groups. Peak immune responses were detected in the spleen and RLN on day 7, which typically corresponds to peak immune responses induced by vaccination [43]. At this time, antigen-specific immune response could also be detected in the LLN, but these responses declined significantly by day 14, especially for the DPX-R9F group. This was interesting because the tumors in the DPX-R9F group were being controlled at this time point and the LLN were small, whereas tumors were not being well controlled in the w/o-R9F group.

This could indicate that the IFN-γ responses are not painting a full picture of the complete immune response occurring at the site of the lymph node. It more likely suggests that activated T cells are actively exiting immune organs and trafficking to the tumor itself, exerting the increased tumor control seen in the DPX vaccinated group as compared to mice vaccinated with the w/o emulsion. We have previously demonstrated that repeated immunizations with emulsion vaccines leads to accumulation of regulatory T cell responses, while DPX formulations do not [37]. In addition, work from other groups [44] has indicated that emulsion vaccines create an ‘antigen sink’ whereby the depot of antigen at the site of injection actively pulls T cells from the systemic circulation and prevents those cells from engaging tumor targets. The lower ELISPOT responses at day 14 suggest that DPX acts less as an antigen sink than w/o emulsions. This is further confirmed in a clinical trial in advanced ovarian cancer patients with DPX-Survivac [35], which produced robust vaccine-induced antigen-specific T cells which persisted systemically for several months, in direct contrast with the observations made with emulsion vaccines in mouse models by others.

This data further underscores the importance of using optimized vaccine platforms for immunization with peptides, and using multiple tests to assess immune responses in the clinic. For example, while the RLN increases in size and cell counts from days 7 to 14, the ELISPOT results decrease. In the LLN, the DPX vaccine does not cause an increase in cell count (and the immune response decreases), but the w/o emulsion causes a strong increase in cell numbers and only a slight increase in IFN-γ specific to DC-R9F and to C3 cells. Increased cell numbers are linked to lymph node swelling but do not appear to be directly correlated with IFN-γ specific responses. This again demonstrates that the complex immune response is not being accurately represented by ELISPOT tests, which is a commonly used measure of immune efficacy in cancer immunotherapy clinical trials.

Of particular interest however was the fact that there did not appear to be any significant differences between DPX and w/o emulsion immune responses, and the emulsion response in fact generally trended higher than DPX. This, however, is in contrast to the actual efficacy results of the vaccine seen by the end of the study, where only 21% of the w/o emulsion group had a positive response to the vaccine compared to 55% of the DPX group. These results indicate that this particular ELISPOT test evaluating the IFN-γ responses from antigen specific T cells does not necessarily predict the eventual efficacy of the vaccine as opposed to our LN biomarkers which, while only measuring volumetric changes, may actually be more representative of the overall immune response.

### Generalizability of the biomarkers to other depot vaccines

It is also important to note that both biomarkers worked not only for one vaccination type (DPX, Supplementary Figure 1), but were also generalizable to another vaccine type, a more conventional water in oil emulsion (w/o emulsion, Figure 3). Although the w/o emulsion was able to cause tumor suppression, it had an overall lower positive final suppression compared to DPX, yet still resulted in significant RLN swelling and strong predictive biomarker responses. Both the % RLN increase and RLN/LLN ratio ROC curves for the w/o emulsion generated very strong AUC values (AUC > 0.8) indicating strong predictive power.

A similarity in critical thresholds is also helpful, although not required, for generalization to multiple vaccination types. For the % RLN increase, the critical cut-off value (whereby any % increase higher than the threshold is “success”) for both DPX and w/o emulsion were quite similar at the day 15 time point. However, for the RLN/LLN ratio, although the thresholds were similar at day 15, by day 22 the thresholds had started to differ more significantly (1.51 *vs* 0.98). This is likely due to two effects: 1) decreased positive responders and 2) increased LLN swelling in the w/o emulsion group. Due to the decreased number of positive responders for w/o emulsion, there are not large differences in sensitivity and specificity when the ratio is modified. For example, when the critical threshold is changed from 1.51 to 0.91, the sensitivity does not change and the specificity only drops from 77% to 71%. The increased LLN swelling in the w/o emulsion group is also an underlying cause of an increased cut-off threshold. As seen in Figure 1, although there was significant tumor suppression in the w/o emulsion group compared to controls, there was more apparent LLN swelling than for DPX mice (although the difference was not significant).

The ROC curves generated by incorporating all mice (Figure 5) do indicate that both biomarkers can be used as accurate prognostic tools for both these vaccine types without separating the groups, although it does result in a decrease in the overall prognostic power of the biomarker (represented in this case by the AUC, which decreased from > 0.8 to between 0.76 and 0.79).

Both biomarkers offer valuable tools for evaluating individualized therapy responses at the preclinical level without sacrificing the mice to obtain more invasive immune responses. The % RLN increase is particularly useful as it offers an early indication of therapy response, between one to two weeks post-vaccination, as opposed to needing to wait until the end of the tumor challenge. These biomarkers are also potentially translatable to other peptide-based vaccines as well offering them wide applicability as a preclinical prognostic tool.

### Clinical translation of lymph node biomarkers

Lymph node swelling is a common physiological response for many clinical vaccines [45, 46]. Results from the current study indicate marked increases in LN volume in response to DPX-R9F and w/o-R9F vaccination - likely an effect that will translate into clinical populations. Both biomarkers are easy to obtain with MRI, or potentially computed tomography (CT) imaging, depending on the LNs of interest. Many clinical trials and patient therapy have diagnostic imaging as a standard of care and if not already done, these scans can easily be added to treatment plans. The RLN/LLN biomarker is useful in that it only requires a single scan session, however even the % RLN increase biomarker only requires one additional scan done prior to treatment (and pre-treatment scans are also typical for standard of care).

The other important issue to consider for clinical translation is the specificity and sensitivity of our potential biomarker. In this work we mathematically determined the critical threshold for success by optimizing the combination of the sensitivity and specificity using Youden's J index. Using this method the RLN/LLN (Day 22) had lower sensitivity (93%) than %RLN (Day 15, 97%), but much better specificity (71% *vs* 55%). However, this may not be the optimal metric for determining the cut-off threshold in a clinical population. It may be better to choose a metric that gives stronger weight to the specificity over sensitivity. We must first determine the clinical responses and then the appropriate cut-off could be decided in consultation with oncologists.

One potential confounding issue for the use of the RLN/LLN ratio is identification of the optimal “LLN”. The clinical equivalent to “RLN” can more generically be defined as “vaccine-draining lymph node”, however this study's “LLN” is more complex, in that it is both the equivalent contralateral LN and also the tumor draining LN. That particular combination is unlikely to be clinically obtainable. In many clinical situations, the tumor draining LN is known and could potentially be used as the “LLN” in this ratio. However, careful consideration and re-evaluation of critical thresholds and resultant ROC curves would be required to determine its predictive power. Given these issues, and the earlier changes in RLN swelling, the % RLN increase biomarker is likely the more clinically relevant choice.

It is also important to note that established tumors in humans are far more complex than experimentally induced tumors in mice. Human tumors, often developing after years of slow growth and active immune evasion, often develop unique inhibitory mechanisms that can dramatically influence the success of an immunotherapy at the tumor level. However, the data presented is valuable in that it establishes that the lymph node swelling functions as a potential biomarker of a “functional” T cell response, with the potential to overcome immune suppressive environments and contribute to tumor growth control, rather than simply a marker of an immune response in general.

The applicability of this technique for predicting clinical success will need further investigation and validation in clinical studies. It is possible that the utility of this technique could be limited to predicting the response of patients to vaccination irrespective of the final effect of the vaccine on the tumor. Patients who fail to mount an immune response following vaccination are unlikely to have a positive clinical response to treatment. In this scenario, this tool can be applied for personalized medicine; a patient who fails to demonstrate immune responsiveness could be promptly switched to another therapy. Rather than simply replace currently utilized immune monitoring assays, such as ELISPOT on peripheral blood T cells, this assay could compliment these assays to provide a more complete picture of patient responses to this complicated treatment.

## CONCLUSIONS

In many clinical trials and in general patient treatment, early evaluation of therapy success currently relies on blood tests measuring systemic immune responses and antigen levels, or changes in tumor volumes such as RECIST, irRC, etc. The biomarker proposed here, % increase in the vaccine draining lymph node (RLN), is a personalized response that could potentially identify which patients are responding early and strongly to therapy, resulting in more efficient clinical development of novel cancer therapeutics.

## MATERIALS AND METHODS

### Cell lines

The C3 cell line (obtained from Dr. Martin Kast) [47] was maintained in Iscove Modified Dulbecco's Medium (IMDM; Sigma, St. Louis, MO) supplemented with 10% heat-inactivated fetal calf serum (Sigma, St. Louis, MO), 2mM L-glutamine (Gibco, Burlington, ON), 50mM 2-mercaptoethanol (Gibco, Burlington, ON), 100U/ml penicillin and 100μg/ml streptomycin (Gibco, Burlington, ON). Cells were incubated at 37°C and 5% CO_2_.

### Peptides

All peptides were synthesized by NeoMPS at > 90% purity. The CD8 epitope HPV16E7_49-57_ (RAHYNIVTF; R9F) and the universal T helper peptide TT_830-843_ (FNNFTVSFWLRVPKVSASHLE; F21E), were used in vaccine formulations.

### Vaccine formulations

Vaccines were prepared either as a proprietary DPX formulation [37, 43] or using a water-in-oil (w/o) emulsion [48]. For DPX with R9F, lipid-mixture containing phosphotidyl choline and cholesterol in a 10:1 ratio (w:w) (Lipoid GmBH, Germany), R9F (5 μg/dose), F21E (5 μg/dose), and a proprietary polynucleotide based adjuvant (20 μg/dose) were formulated in 40% tert-butanol, freeze-dried and resuspended in Montanide ISA51 VG (SEPPIC, France). For DPX no R9F group, followed similar procedure as described above without adding R9F and F21E to the formulation. Water-in-oil emulsion were prepared by mixing R9F (5 μg/dose) and F21E (5 μg/dose) in sterile water, followed by mixing the prepared antigen solution with equal volume of Montanide ISA51 VG to form a homogeneous emulsion.

### Tumor challenge and vaccination

C57BL/6 female mice (4-6 weeks old, pathogen free) were obtained from Charles River Laboratories (St. Constant, PQ) and housed with food and water *ad libitum* under filter top conditions. Experiments involving the use of mice were carried out in accordance with protocols approved by the University Committee on Laboratory Animals at Dalhousie University, Halifax, N.S., Canada.

For the imaging experiment, 100 mice underwent C3 tumor cell implantation, with 5×10^5^ cells implanted subcutaneously (s.c.) into the left flank (Study day −5). Five days post-implantation (Study Day 0), mice received either i) DPX-R9F: 50 μL of DPX with R9F and F21E (*n* = 45), ii) DPX (no R9F): 50 μL of DPX with no R9F or F21E (*n* = 7), iii) w/o-R9F: 100 μL of W/O emulsion with R9F and F21E (*n* = 29), or iv) PBS control injection (*n* = 19). Vaccine formulations were delivered *via* a single s.c. contralateral immunization (right flank). Tumor sizes were determined every few days with calipers using the following formula: longest measurement x (shortest measurement)^2^ divided by 2.

### IFN-γ ELISPOT

In order to evaluate immune responses, 40 mice underwent C3 tumor cell implantation and vaccination as described above. IFN-γ ELISPOT assay was performed as previously described [43]. Briefly, mature dendritic cells (DCs) were generated by culturing bone marrow cells from naive C57BL/6 mice in complete RPMI media [RPMI 1640 (Gibco),10% FBS (Hyclone), <2% penicillin/streptomycin (Gibco), 2mM L-glutamine (Gibco), 50 mM β-mercaptoethanol (Sigma-Aldrich), and 5mM HEPES buffer (Gibco)] supplemented with murine GM-CSF (Peprotech). DCs were loaded with 10 μg/mL peptides on day 7. Day 8 DCs were resuspended in complete RPMI at 2 × 10^5^ cells/mL and used as antigen presenting cells for the ELISPOT assay.

Both right and left inguinal lymph nodes were collected from mice upon euthanasia. Single cell suspensions were prepared in complete RPMI media and cell concentration adjusted to 2 × 10^6^ cells/mL. Lymph node cells (100 μL) and DCs (100 μL) were added to IFN-γ ELISPOT plates (BD Bioscience). The ELISPOT plate was incubated overnight at 37°C, 5% CO_2_ and then developed the next day using AEC substrate kit (Sigma-Aldrich). Spots were counted using an ImmunoSpot Analyzer, ELISPOT plate reader (C.T.L. Ltd, Shaker Heights, OH, U.S.A.) and enumerated as number of spot-forming units (SFU) per well.

IFN-γ ELISPOT performed using splenocytes had the following modifications: Single cell suspensions of splenocytes were prepared by lysing red blood cells with ammonium-chloride-potassium solution and resuspending the cells at 5 × 10^6^ cells/mL in complete RPMI media. A volume of 100 μL cells was added into IFN-γ ELISPOT plate and stimulated with 100 μL complete RPMI containing no peptide (background control), 20 μg/mL R9F or irrelevant peptide, or 5 × 10^5^ cells/mL C3 tumor cells.

### Data acquisition and MR imaging

All data were acquired on a 3T magnet equipped with 21 cm ID gradient coil (Magnex Scientific, Oxford, UK) interfaced with a Varian DD Console (Varian Inc., Palo Alto, Ca). A 30mm ID quadrature transmit/receive RF coil (Doty Scientific, Col., SC), was used to image tumors, vaccination sites, and left & right inguinal lymph nodes simultaneously.

MRI scans were performed between Days 0-3 and then weekly for 6 weeks to evaluate tumor progression/eradication as well as lymphatic response. Baseline scans were also performed prior to tumor challenge (Day −13) to allow proper comparison of anatomical structures, for a total of 7 MRI time points in the study.

Sagittal images were obtained using a 3D balanced steady-state free precession (bSSFP) sequence with a repetition time (TR)/echo time (TE) = 8/4 ms, flip angle = 30°, a 38.4×25.5×25.5 mm field of view (FOV) with a 256×170×170 matrix centred on the torso, giving voxels with 150μm isotropic resolution. 6 signal averages were acquired with two frequencies [49] for a total scan time of approximately 48 minutes per animal.

### MRI image analysis

Volumetric segmentation of structures were performed by a single observer, in a blinded fashion to eliminate the prospect of observer bias, and were then confirmed by a second independent reviewer. All images were first zero-padded (interpolated to a higher resolution grid to increase the effective resolution and image quality) using ImageJ (NIH). Images were analyzed in RView for each mouse [50, 51]. A semi-automated region growing algorithm was implemented to perform individual 3D segmentations to determine i) C3 tumor volumes, ii) left inguinal lymph node (LLN), and iii) right inguinal lymph node volumes (RLN).

### Statistical analysis

Statistical comparisons of each of the aforementioned volumetric variables measured and the RLN/LLN ratio were made using a mixed analysis of variance (ANOVA) to assess main effects, followed by Games-Howell post-hoc tests (due to unequal variances between groups) to assess statistical significance (corrected *p* < 0.05). The mixed ANOVA assesses differences both between groups (between-subjects factor) and within groups over time (within-subjects factor). All statistical analyses were done using SPSS v22 (IBM). All data are presented as group means ± SEM.

### Receiver operating characteristic (ROC) curves

LN volumetry and associated metrics were assessed using receiver operating characteristic (ROC) curves [52]. Tumor suppression was judged at the end of the study, 6 weeks post-implantation. Tumor suppression was found to be successful (i.e. “positive”) if there was no tumor left, or if the tumor remaining was judged to be “palpable”, defined as present but with volume < 50mm^3^. The false positive fraction (FPF) was calculated as FP/(FP+TN) and the true positive fraction (TPF) was calculated as TP/(TP+FN) at each week of the study and they were compared in ROC space using both “ROC-KIT” ROC analysis software [53–56], and SPSS (IBM SPSS Statistics for Macintosh, Version 22.0). ROC curves (sensitivity *vs*. 1-specificity) were generated at each week of the study using a number of potential imaging biomarkers described in the results. ROC curves were generated for five time points, day 2, day 8, day 15, day 22, and day 29 post-treatment. Both the % increase in RLN volume and the volumetric ratio of RLN/LLN were evaluated as potential biomarkers. We evaluated ROC curves in three groups/subsets: 1) mice receiving only DPX-R9F or control injection (both PBS and DPX with no R9F), 2) mice receiving only w/o-R9F or PBS injection, or 3) all mice.

The area under the ROC curve (AUC) is a common summary measure of a diagnostic test's performance, interpreted as the average sensitivity for all possible values of specificity [52]. AUC represents the overall performance and diagnostic accuracy of a test, with values approaching 1, indicating perfect accuracy. AUC was measured from the fitted data. In order to evaluated the critical threshold for optimizing biomarker success, Youden's J statistic (J = Sensitivity + Specificity − 1), a tool that is often used for evaluating a diagnostic test [41, 57], was calculated and maximized for those ROC curves where the AUC > 0.7. The critical threshold and corresponding sensitivity and specificity were calculated for the maximized J value for each curve using SPSS.

## SUPPLEMENTARY MATERIAL FIGURE


