# Supplementary material for: Using lymph node swelling as a potential biomarker for successful vaccination
Source: Oncotarget. 2016 May 24;7(24):35655–69. doi: 10.18632/oncotarget.9580 (PMC5094952; doi:10.18632/oncotarget.9580)
Supplement: Supplementary file 1 [file oncotarget-07-35655-s001.pdf]

Using lymph node swelling as a potential biomarker for successful vaccination

Supplementary Material

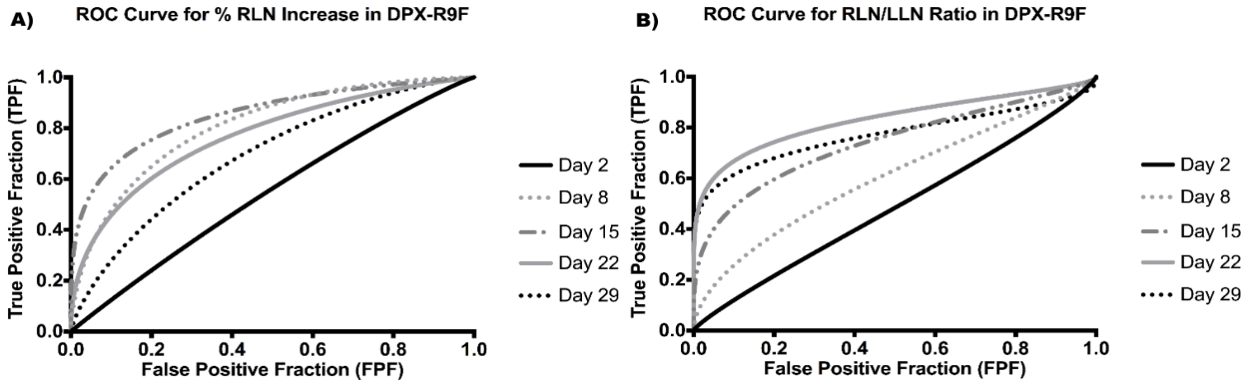

C)

	% RLN	RLN/LLN
**Day 2**	0.544 ± 0.091	0.489 ± 0.000
**Day 8**	0.807 ± 0.054	0.606 ± 0.067
**Day 15**	0.853 ± 0.046	0.742 ± 0.058
**Day 22**	0.769 ± 0.057	0.831 ± 0.049
**Day 29**	0.689 ± 0.073	0.772 ± 0.065

Supplementary Figure 1 – ROC Curves for Biomarkers in DPX group. A) Fitted ROC curves for each time point for % RLN increase biomarker. B) Fitted ROC curves for each time point for RLN/LLN ratio biomarker. C) Area under the curve (AUC) values for each biomarker at each time point. Optimal values are highlighted.
